# Treatment of acute coronary syndrome by ground and helicopter ambulance services in Poland: a five-year retrospective study

**DOI:** 10.3389/fpubh.2026.1777641

**Published:** 2026-05-14

**Authors:** Piotr Konrad Leszczyński, Arkadiusz Wejnarski, Weronika Buźniak, Tomasz Ilczak, Kryspin Mitura, Krzysztof Marek Mitura, Daniel Celiński, Sławomir Dariusz Szajda

**Affiliations:** 1 Department of Medical and Health Sciences, University of Siedlce, Siedlce, Poland; 2 Department of Health Sciences, University of Bielsko-Biala, Bielsko-Biała, Poland; 3 Independent Public Health Care Center RM-MEDITRANS Emergency Station and Sanitary Transport in Siedlce, Siedlce, Poland; 4 Department of Emergency Medical Service, Medical University of Warsaw, Olsztyn, Poland; 5 Department of Emergency Medical Service, Collegium Medicum, University of Warmia and Mazury in Olsztyn, Olsztyn, Poland

**Keywords:** acute coronary syndrome, emergency medical service, helicopter emergency medical service, out-of-hospital cardiac arrest, physician, treatment

## Abstract

**Background:**

Acute coronary syndrome (ACS) is a serious health problem, sometimes directly threatening a patient's life. According to current medical knowledge, appropriate management should be implemented as early as in the prehospital settings. The authors conducted an analysis aimed at comparing EMS (with physician) and HEMS interventions over a five-year period in patients with suspected or diagnosed acute coronary syndrome. The objective of the study was to outline the profile of a patient with ACS who requires assistance in pre-hospital conditions.

**Methods:**

87,716 interventions by ground teams (EMS–emergency medical service) and 3,045 by air teams (HEMS–helicopter emergency medical service) were included in the study. The analysis covered the period from 2018 to 2022, including nationwide data made available from information technology systems. The material was selected from an extensive collection using the International Classification of Disease Diagnoses, ICD-10: I21 (I21.0, I21.1, I21.2, I21.3, I21.4, and I21.9). Statistical design was used, allowing for correlations of variables, at a significance level of *p* < 0.05.

**Results:**

The majority of the subjects were women (65.2%), and the mean age was 65.94 years (SD ± 12.30; Min: 13 years old; Max: 126 years old). In both ground team patients (95.7%) and helicopter team patients (98.2%), the predominant mechanism of OHCA was non-defibrillatory rhythm (PEA/asystole). In airborne teams, external electrical stimulation, bladder catheterization and gastric tube placement were implemented significantly more often than in ground teams (*p* < 0.001). An evaluation of pharmacotherapy with opioid agents showed relatively frequent administration of morphine (EMS: 33.2%; HEMS: 20.1%), and occasional administration of fentanyl (EMS: 4.7%; HEMS: 6.1%). Both types of teams occasionally (EMS: 16.4%; HEMS: 1.9%) administered Clopidogrel, with Ticagrelor being more frequent (EMS: 21.2%; HEMS: 6.5%).

**Conclusions:**

There are significant differences in the behavior of EMS and HEMS physicians. Future studies are worth considering to understand the correlation between the performance of these procedures by EMS physicians before hospitalization.

## 1 Introduction

Acute coronary syndrome (ACS) according to the World Health Organization (WHO) is a clinical condition resulting from a sudden and critical restriction of blood flow through the blood vessels of the heart, leading to myocardial infarction (MI) (1). Myocardial cell damage during ACS can take different forms, depending on the degree of ischemia and the duration of the condition. There are three main types:

- unstable angina, in which there are no characteristic changes in the form of ST-segment elevation on the electrocardiogram (ECG), while elevated levels of cardiac biomarkers such as troponin are observed in laboratory results;
- non-ST-segment elevation myocardial ischemia (NSTEMI) ECG shows no ST-segment elevation, but other changes, such as ST-segment depressions or T-wave inversions, may be evident, while laboratory tests show elevations in cardiac biomarkers;
- ST-segment elevation myocardial ischemia (STEMI) is the most serious type of ACS, the ECG shows marked ST-segment elevation, and laboratory tests usually show significantly elevated troponin levels (2, 3).

Diagnosis and treatment should be started as soon as possible due to the progressive necrotic lesions. The latest European Society of Cardiology guidelines for the management of acute coronary syndromes were published in 2023. Diagnostics are based on ABCDE assessment, physical examination and SAMPLE history (4). The target treatment is percutaneous coronary interventions (PCI), but appropriate pharmacological management should be implemented as early as at the prehospital stage (5, 6). In addition to analgesic treatment (mainly opioids), heparin, acetylsalicylic acid (ASA) and an adenosine 5′-diphosphate (ADP) receptor inhibitor, which is a P2Y12 receptor antagonist, should be considered in the absence of contraindications. According to data from the National Health Fund in Poland, the mortality rate in diagnosed ACS reached up to 78% (2014).

The procedure described above, depending on the country's emergency medical system, is implemented as early as at the pre-hospital stage. In Europe, ground medical emergency teams (EMS) and air medical rescue teams (HEMS)–formed by PMAR (Polish Medical Air Rescue) in Poland–are most often dispatched to the scene of life-threatening emergencies (7–9). Air teams consist of a doctor, a paramedic/nurse, and a pilot (10, 11). The medical staff in more than 80% of the ground teams in Poland consists of paramedics and nurses, with a doctor leading the rest of the units. Depending on the type of airborne EMS, some medical procedures and the set of medications in the inventory vary (12, 13). Nevertheless, each team is equipped with the necessary equipment to diagnose a cardiac patient and implement initial treatment, for the duration of transport to the hospital (14). Both HEMS teams and EMS teams with a physician have the same competences and diagnostic and therapeutic capabilities.

The authors, knowing the specifics of the work of ground and air medical rescue teams in Poland, conducted an analysis aimed at comparing EMS and HEMS interventions over a five-year period in patients with suspected or diagnosed acute coronary syndrome. The specific objective of the study was to outline the profile of a patient with ACS who requires assistance in pre-hospital conditions. Medical procedures and pharmacotherapy implemented by EMS and HEMS physicians were determined, conducting a comparative analysis on a national scale.

## 2 Materials and methods

### 2.1 Material

The study material consisted of data from the medical records of ground emergency medical teams (EMS) and the base of the Airborne Emergency Medical Services (PMAR). Nationwide data in digital format covered a five-year period (2018–2022). At the request of the research team and with the approval of the Ministry of Health in Poland, anonymized data from the Command Support System of the National Emergency Medical Service was made available. Analogous approvals were obtained from PMAR management. At the time of the study, the number of HEMS bases in Poland is 22, and the number of ground medical rescue teams is 1617 (including only 315 with physician). In the years 2018–2022, the number of teams was smaller and varied from year to year. Therefore, the authors, when making comparative analyses, took into account the percentage ratio in the individual study groups (EMS and HEMS). The total number of cases analyzed was 90,761.

Formal procedures allowed us to obtain data from both databases (EMS and HEMS), containing interventions from all over the country, which allowed for full representativeness of the studied population in Poland. The inclusion criterion was the inclusion of the verbal diagnosis of ACS (and suspicion of ACS) in the medical documentation, as well as the inclusion of a code according to the international classification of diseases ICD-10, such as: I21 (I21.0, I21.1, I21.2, I21.3, I21.4, and I21.9). The diagnoses were made by emergency medical team physicians. They were not linked to further in-hospital procedures. Digital medical documentation requires entering key information after each intervention, therefore the data obtained was complete. The authors assessed only the calls of teams that included a physician. This allowed for an additional comparison of medical interventions undertaken by air and ground rescue teams, because their competencies are the same. The study received a positive opinion from the Research Ethics Committee, No. 2/2024, dated January 18, 2024.

### 2.2 Methods

The data extracted from the two databases was organized in spreadsheets, allowing further statistical analysis. The significance level was set at *p* = 0.05, understood as the maximum acceptable probability of error, causing the rejection of the true hypothesis. According to the above, results with *p* < 0.05 were considered as demonstrating presence of significant relationships between variables. Variables expressed at the ordinal or nominal level were analyzed using tests based on chi-square distribution. For 2 × 2 tables, a continuity correction was applied, while when the conditions for using the chi-square test were not met, Fisher's exact test with expansion for tables larger than 2 × 2 was used. Non-parametric tests (Mann-Whitney U test) were used to analyze quantitative variables presented by group. The tests were selected based on the distribution of variables, which was verified by the Kolmogorov-Smirnov test. Calculations were performed in the statistical environment of R ver.3.6.0, the PSPP program and MS Office 2019.

## 3 Results

### 3.1 Characteristics of the study group

The study group consisted of patients with suspected or diagnosed acute coronary syndrome from all over Poland to whom EMS or HEMS was dispatched between 2018 and 2022. The group can be differentiated by sociodemographic factors. The majority of the subjects were women (65.2%), and the mean age was 65.94 years (SD ± 12.30; Min: 13 years old; Max: 126 years old). EMS was ordered significantly more often (*n* = 87,716; 96.6%). Considering the number of EMS and HEMS calls, the largest number of interventions occurred in 2018 (*n* = 19,013; 20.9%). A significant number of cases occurred during the non-pandemic period (*n* = 50,454; 55.6%). The characteristics of the study group along with the ICD-10 diagnosis are presented in Table 1. According to the ICD 10 classification, I21 (acute myocardial infarction) accounted for the largest number of cases, as well as I21.9 (acute myocardial infarction, unspecified) and I21.1 (acute full-thickness myocardial infarction, inferior wall). I22.8 was the rarest diagnosis in this group (recurrent myocardial infarction of another location).

**Table 1:** Characteristics of the study group.

Date		*N*	*%*
Medical team	EMS	87,716	96.6%
	HEMS	3,045	3.4%
Year	2018 (EMS = 18,292; HEMS = 721)	19,013	20.9%
	2019 (EMS = 17,760; HEMS = 698)	18,458	20.3%
	2020 (EMS = 16,732; HEMS = 588)	17,320	19.1%
	2021 (EMS = 17,711; HEMS = 508)	18,219	20.1%
	2022 (EMS = 17,221; HEMS = 530)	17,751	19.6%
Period	COVID-19 pandemic	40,307	44.4%
	Beyond the COVID-19 pandemic	50,454	55.6%
Gender	Female	58,589	64.5%
	Male	31,292	34.5%
	No gender determination	880	1.0%
ICD-10 diagnosis	I21	44,267	48.8%
	I21.0	5,573	6.1%
	I21.1	10,553	11.6%
	I21.2	1,040	1.1%
	I21.3	485	0.5%
	I21.3	608	0.7%
	I21.9	27,463	30.3%
	I22	585	0.6%
	I22.0	95	0.1%
	I22.1	146	0.2%
	I22.8	38	0.0%
	I22.1	166	0.2%

The analysis of the teams' interventions included the critical period of the COVID-19 pandemic, which significantly affected health systems around the world (15, 16). For ground teams, it was the period from 03.2020 to 05.2022 (*n* = 38,681; 44.1%), and for air teams, it was 2020–2022 (*n* = 1,626; 53.4%). The result was statistically significant (χ^2^ = 10.366; *df* = 3; *p* = 0.016). This shows that there are large differences in the distribution of calls across periods by type of team. Ground teams intervened significantly most often outside the pandemic (n = 49,035; 55.9%), and air teams in the pandemic (*n* = 1,626; 53.4%). A significant relationship (χ^2^=1,774.525; *df* = 1; *p* < 0.001) was also observed in the sex distribution in terms of the emergency team type. EMS most often provided medical aid to women (*n* = 57,694; 66.4%) and HEMS most often provided medical aid to men (*n* = 2,147; 70.6%). In half of the ground team's calls, patients were no younger than *Me* = 66.00 years (in the other half, no older than *Me* = 66.00). In half of the airborne team's calls, patients were no older than *Me* = 65.00 (in the other half, no younger than *Me* = 65.00). When assessing this parameter, the normality of variable distribution was checked using the Kolmogorov-Smirnov test, the result of which indicated the validity of using a non-parametric test (Mann-Whitney U-test) comparing the medians of the dependent variable across groups. It was demonstrated that ground teams provided aid statistically significantly more often (*p*<*0*.001) to older patients than air teams.

### 3.2 Sudden cardiac arrest

Among the ground teams, a statistically significant result (*p* = 0.016) was obtained, indicating large differences in the distribution of OHCA (out-of-hospital cardiac arrest) incidence by age. In case of ground-based emergency teams, OHCA was significantly more common among patients aged 56 to 65 than among those over 75. A similar relationship was not demonstrated for HEMS patients. A detailed breakdown of the number of OHCA cases by type of emergency medical team is presented in Table 2. There was a statistically significant difference (Mann-Whitney test; *p* = 0.02) between the percentage of OHCA cases between EMS and HEMS teams.

**Table 2:** OHCA cases by type of emergency medical team.

Year	*EMS [N] (%)*	*HEMS [N] (%)*
2018	1,251 (6.84%)	21 (2.91%)
2019	1,305 (7.35%)	18 (2.58%)
2020	1,140 (8.61%)	22 (3.74%)
2021	1,105 (6.24%)	28 (5.51%)
2022	936 (5.44%)	16 (3.02%)
Total	**5,737**	**105**

An analysis of cardiac rhythm as a mechanism of cardiac arrest according to medical services team type was made. Patients in case of both ground-based and airborne teams had non-defibrillatory rhythm (PEA/asystole) as the predominant mechanism of OHCA. When comparing groups of EMS vs. HEMS patients, it was demonstrated that defibrillation rhythm (VF/pVT) was diagnosed significantly more often among ground team patients (χ^2^ = 40.451; *df* = 1; *p* < 0.001).

### 3.3 Medical procedures

Standard treatment for every patient diagnosed with ACS includes, among others, placement of a venous catheter, ECG, monitoring, blood pressure measurement, etc. Other procedures were selected, which are implemented depending on the severity of the patient's condition with ACS. Statistically significant results were obtained for the implementation of external electrical stimulation, bladder catheterization, insertion of a gastric tube, intubation and mechanical ventilation, showing significant differences in their distribution by medical team type. Only electrical cardioversion use yielded a statistically insignificant result. The data show that the following were implemented significantly more often in airborne teams than in ground teams: external electrical stimulation, bladder catheterization and insertion of a gastric tube. In contrast, the use of endotracheal intubation was not significantly dependent on the type of team (Table 3).

**Table 3:** Medical procedures performed by EMS and HEMS.

Treatment			Medical team		Test results
			EMS	HEMS	
Cardioversion	No	N	87,520	3,039	*χ^2^* = *0.012; df* = *1; p* = *0.914*
		%	99.8%	99.8%	
	Yes	N	196	6	
		%	0.2%	0.2%	
External pacing	No	N	87,428	3,019	*χ^2^* = *22.074; df* = 1; *p* < 0.001
		%	99.7%	99.1%	
	Yes	N	288	26	
		%	0.3%	0.9%	
Bladder catheterization	No	N	87,601	3,007	*χ^2^* = *211.532; df* = 1; *p* < 0.001
		%	99.9%	98.8%	
	Yes	N	115	38	
		%	0.1%	1.2%	
Gastric tube	No	N	87,486	3,028	*χ^2^* = *8.446; df* = 1; *p* = *0.004*
		%	99.7%	99.4%	
	Yes	N	230	17	
		%	0.3%	0.6%	
Intubation	No	N	83,749	2,933	*χ^2^* = *4.694; df* = 1; *p* = *0.030*
		%	95.5%	96.3%	
	Yes	N	3,967	112	
		%	4.5%	3.7%	
Ventilation	No	N	83,326	2,948	*χ^2^* = *20.340; df* = *1; p < 0.001*
		%	95.0%	96.8%	
	Yes	N	4,390	97	
		%	5.0%	3.2%	

χ2, statistical test; *df* , degrees of freedom; *N*, number; *p*, statistical significance.

### 3.4 Drug treatment

One of the key measures is analgesic treatment of the ACS patient, who usually presents with significant pain, which is the first recognizable symptom. Two opioid drugs are available to medical personnel in EMS and HEMS teams in Poland: fentanyl and morphine. An evaluation of pharmacotherapy with opioid agents showed relatively frequent administration of morphine (EMS: 33.2%; HEMS: 20.1%), and occasional administration of fentanyl (EMS: 4.7%; HEMS: 6.1%). It was proven that air teams administered fentanyl statistically significantly more often, with morphine being administered less often to ACS patients, compared to ground teams (*p* < 0.001) (Table 4).

**Table 4:** Pharmacotherapy dedicated to patients with ACS by EMS and HEMS.

Treatment			Medical team		Test results
			EMS	HEMS	
Fentanyl	No	N	83,634	2,860	*χ^2^* = *12.964; df* = *1; p < 0.001*
		%	95.3%	93.9%	
	Yes	N	4,082	185	
		%	4.7%	6.1%	
Morphine	Yes	N	29,126	611	*χ^2^* = *230.029; df* = *1; p < 0.001*
		%	33.2%	20.1%	
	No	N	58,590	2,434	
		%	66.8%	79.9%	
Ticagrelor	Yes	N	18,625	197	*χ^2^* = *389.336; df* = *1; p < 0.001*
		%	21.2%	6.5%	
	No	N	69,091	2,848	
		%	78.8%	93.5%	
Clopidogrel	Yes	N	14,399	59	*χ^2^* = *459.521; df* = *1; p < 0.001*
		%	16.4%	1.9%	
	No	N	73,317	2,986	
		%	83.6%	98.1%	

χ^2^, statistical test; *df* , degrees of freedom; *N*, number; *p*, statistical significance.

The Polish emergency medical system does not provide targeted reperfusion therapy procedures. Prehospital pharmacotherapy involves oral and intravenous medications. Heparin was administered 45,276 times by ground teams in the study group, and 343 times by air teams. P2Y12 receptor antagonist drugs, such as Ticagrelor and Clopidogrel, are part of both HEMS and EMS team inventory. Both types of teams occasionally (EMS: 16.4%; HEMS: 1.9%) administered Clopidogrel, with Ticagrelor being more frequent (EMS: 21.2%; HEMS: 6.5%). A significant statistical difference was shown by analyzing the supply of this group of drugs comparing the two teams, as shown in Table 4. EMS administered P2Y12 receptor antagonist drugs significantly more often than HEMS.

Patients with circulatory and/or respiratory failure require additional management (e.g., endotracheal intubation with muscle relaxants, administering drugs affecting adrenergic and dopaminergic receptors). A group of seven substances is listed, which are available in both types of medical teams. An analysis of the implemented pharmacotherapy shows that air teams were significantly more likely to administer all of the listed medications to patients (Table 5).

**Table 5:** Muscle relaxants and adrenergic and dopaminergic drugs administered by EMS and HEMS.

Treatment			Medical team		Test results
			EMS	HEMS	
Chlorsuccillin (suxamethonium chloride)	No	N	87,655	3,029	*χ^2^* = *66.882; df* = *1; p < 0.001*
		%	99.9%	99.5%	
	Yes	N	61	16	
		%	0.1%	0.5%	
Rocuronium bromide	No	N	87,658	2,960	*χ^2^* = *1,372.221; df* = *1; p < 0.001*
		%	99.9%	97.2%	
	Yes	N	58	85	
		%	0.1%	2.8%	
Dobutamine (dobutamine hydrochloride)	No	N	87,677	3,017	*χ^2^* = *293.749; df* = *1; p < 0.001*
		%	100.0%	99.1%	
	Yes	N	39	28	
		%	0.0%	0.9%	
Dopamine (dopamine hydrochloride)	No	N	87,094	3,013	*χ^2^* = *4.340; df* = *1; p* = *0.037*
		%	99.3%	98.9%	
	Yes	N	622	32	
		%	0.7%	1.1%	
Ephedrine (ephedrine hydrochloride)	No	N	87,706	3,021	*χ^2^* = *453.663; df* = *1; p < 0.001*
		%	100.0%	99.2%	
	Yes	N	10	24	
		%	0.0%	0.8%	
Levonor (norepinephrine tartrate)	No	N	87,275	3,022	*χ^2^* = *3.211; df* = *1; p* = *0.073*
		%	99.5%	99.2%	
	Yes	N	441	23	
		%	0.5%	0.8%	
Noradrenaline	No	N	87,716	3,007	*χ^2^* = *1,065.492; df* = *1; p < 0.001*
		%	100.0%	98.8%	
	Yes	N	0	38	
		%	0.0%	1.2%	

χ2, statistical test; *df* , degrees of freedom; *N*, number; *p*, statistical significance.

## 4 Discussion

### 4.1 Characteristics of emergency medical team interventions

This paper attempts to analyze the mission of air ambulance services compared to ground medical rescue teams with a doctor. The specifics of the activities of the two types of teams are different, but the diagnostic and therapeutic possibilities are comparable, therefore comparing the cases and medical procedures performed seems justified. The patient profile with suspected or diagnosed ACS was characterized as female (65.2%) with a mean age of 65.94 years (SD ± 12.30). An interesting correlation was identified indicating a different patient profile from the HEMS perspective, wherein the vast majority of missions were performed providing aid to men (n=2147; 70.6%). The prevalence of men with ACS receiving care in the pre-hospital setting was also shown by Rzońca et al. (6), where men accounted for 54.9% of cases. The lack of collation of interventions by all ground teams (including teams without a doctor) can be considered a confounding factor in this correlation. The authors note that in Poland, HEMS deployment often involves assisting the ground team in transporting the patient to a higher referral center more quickly. In such cases, given the patient's poor condition, this is a beneficial solution. It can therefore be assumed that the general condition of men with ACS was worse than that of women, which is why decisions were made to expedite transport to the hospital.

The etiology of OHCA most often involves cardiogenic causes, including acute coronary syndromes (17). ACS, having a varied course, sometimes produces unusual symptoms that may be overlooked or downplayed. In some cases (e.g., NSTEMI ACS), even an ECG test will not be helpful in differential diagnosis of patient's complaints. Progressive ischemia can cause chest pain, circulatory failure and arrhythmia (18, 19). Minimizing the time to PCI, and therefore the role of emergency medical teams, appears to be a key element in the chain of management (20, 21). The obtained results clearly indicated that the cases of resuscitation performed by HEMS teams are significantly less frequent (*p* = 0.02) than in EMS teams. The difference may be due to the earlier arrival of ground EMS at the scene and implementation of resuscitation, which results in the lack of HEMS call for transport to a higher referral center.

Dispatching ground teams is by far more frequent than PMAR helicopters (*n* = 87,716 vs. *n* = 3,045), which is undoubtedly due to at least the much smaller number of HEMS crews compared to EMS. The numbers of teams are variable, but at the time of drafting the article, there were: 22 PMAR helicopter bases and 1617 ground EMS bases in Poland (22). The dispatcher's choice of the appropriate emergency medical unit may also be related to the logistics and availability of teams in a specific area (10).

### 4.2 Pharmacotherapy

Administration of opioid drugs to patients experiencing coronary pain was evaluated during the study period. The results show significant differences in the frequency of fentanyl and morphine administration by ground and air teams. EMS chose to administer fentanyl in only 4.7% of cases and morphine in 33.2%. HEMS personnel administered fentanyl in 6.1% of cases, while morphine was used in 20.1%. Although both types of teams relatively rarely opted for opioid administration in coronary artery pain, the HEMS doctors performed administered fentanyl, which is dedicated in ACS patients, statistically more frequently. It should be assumed that the implementation of periodic training for emergency team physicians may translate into their use of the most up-to-date medical procedures (23–25). In a pilot study, Latos and Ladowska et al. (26) attempted to assess paramedics‘ preferences for administering morphine. At that time, the vast majority (72%) indicated ACS as an indication for the supply of this drug. On the other hand, in a study by Szyller et al. (27), the authors proved that medical personnel choose to administer morphine only when there is no satisfactory response to nitrate drugs. One may wonder why the current beliefs are supported, despite existing evidence and recommending treating pain with a dedicated opioid agent as necessary (4). In their study, Garrick et al. (28) indicate that fentanyl is safer and more effective in pain treatment in the prehospital settings, which does not correlate with doctors' real-world management of coronary pain in 2018–2022 in Poland. In addition, Weldon et al. (29) studied 187 patients with ACS and showed that there was no significant difference in the choice of morphine or fentanyl, given the effect of either drug on changes in blood pressure. The authors of the study point to fentanyl as a good alternative to morphine for patients with out-of-hospital ACS (29).

Another group of drugs analyzed in this study included muscle relaxants and positive inotropic agents. The frequency of using medications such as Chlorsuccillin, Rocuronium bromide, Dobutamine, Dopamine, Ephedrine, Levonor, and Noradrenaline was investigated. The results show a varied approach to using these drugs depending on the type of team, with a clear advantage for HEMS in the frequency of their administration. Chlorsuccillin is a muscle relaxant that works by blocking neuromuscular conduction. It is used in conditions requiring complete muscle relaxation, such as during endotracheal intubation. The study found that both ground (0.1%) and air teams (0.5%) rarely administered this drug. However, helicopter teams did so significantly more often (*p* < 0.001). This may be related to the more frequent need for advanced procedures in air transport conditions. Rocuronium bromide is another muscle relaxant used primarily in anesthesia to facilitate intubation and provide optimal conditions for mechanical ventilation. The study found that ground teams rarely chose to administer the drug (0.1% of cases), while air teams administered it slightly more often (2.8% of cases). This difference may be due to situations requiring advanced airway clearance. Dobutamine is a drug that has a positive inotropic effect, meaning that it increases the force of myocardial contraction. It is often used in cases of acute heart failure, which can accompany ACS. Ground teams chose not to administer Dobutamine in any case (0.0%), while air teams did so in 0.9% of interventions. This may indicate a more restrictive approach to treating heart failure in helicopter teams. Dopamine is another inotropic drug that, in addition to increasing the strength of heart contraction, can also improve blood flow through the kidneys, which is key to treating shock. The study found that ground teams rarely administered the drug (0.7%), while air teams did (1.1%). This difference may be due to the higher number of critical cases that require immediate enhancement of cardiac and renal function under air transport conditions. There is also the possibility of greater restraint in using this pharmaceutical group among EMS doctors, and this requires further studies and objective evaluation of the trend. Ephedrine is a sympathomimetic drug that works by stimulating adrenergic receptors, leading to an increase in the rate and strength of heart contraction, and increasing blood pressure. It is used to treat hypotension, which can occur in ACS. Ground teams chose not to administer the drug in any case (0.0%), while flight teams implemented ephedrine treatment in 0.8% of cases. Similar conclusions were drawn from the use of Levonor as a vasoconstrictor pressor amine for the treatment of severe hypotension and shock, as ground teams administered the drug in only 0.5% of interventions, while airborne teams chose to administer it in 0.8% of cases. Ground teams chose not to administer norepinephrine in any of the cases (0.0%), while the HEMS doctor included the drug in 1.2% of cases. To further analyze the phenomenon, it is necessary to focus on evaluating specific clinical cases to better understand situations in which using selected drugs brings the greatest benefit. It is also worth noting other factors that affect decisions on drug administration, such as the time from the onset of symptoms to the arrival of the emergency medical team, the experience of staff in administering drugs (especially on infusion pumps), the availability of drugs and equipment in different regions of the country, and the individual characteristics of patients, such as age, sex, the presence of other medical conditions, and responses to treatment.

The study also included the use of substances such as Ticagrelor and Clopidogrel, which are oral P2Y12 receptor antagonists. Authors showed a different trend, as EMS administered both Ticagrelor and Clopidogrel significantly more often than HEMS. In the future, it is worth considering further studies to better understand the reasons for these differences and their impact on the outcomes of ACS patients. Certainly, the possibility of using oral medications is affected by the patient's condition. Reduced consciousness level, regurgitation risk, or lack of physiological reflexes may be reasons for withdrawing oral anticoagulant treatment. However, the severity of ACS cases can be indirectly interpreted by the incidence of cardiac arrest. The analysis showed that OHCA was quite common in both types of teams (EMS: *n* = 5,584 [6.90%]; HEMS: *n* = 103 [3.89%]). Nevertheless, it was significantly more frequent (*p* < 0.001) in EMS conditions, suggesting that the condition of patients transported by ground teams was more serious. Despite this, the frequency of oral medication use was higher. It should be noted that only EMS teams with a doctor were included in the analysis. The remaining majority of ground teams is made up of paramedics. There is literature evidence of comparable effectiveness of resuscitation efforts and return of circulation, therefore authors believe that the quality of interventions provided by teams without a doctor is at a similar level in Poland (30, 31).

### 4.3 Medical interventions

The relationship between the type of team (EMS vs. HEMS), and the implementation of selected medical procedures in patients with acute coronary syndrome has been determined. The frequency of using interventions such as electrical cardioversion, electrical stimulation, bladder catheterization, gastric tube placement, endotracheal intubation and mechanical ventilation was studied. Most of the procedures listed (except cardioversion, endotracheal intubation and ventilation) were more often performed by HEMS doctors. While the insertion of a gastric tube and urinary bladder catheterization can be explained by the need to safeguard the patient's physiological reflexes during flight, it is difficult to explain such significant differences in using electrostimulation. Cardioversion and external electrostimulation constitute life-saving procedures for severe arrhythmias in hemodynamically unstable patients. Failure to implement these procedures can result in significant deterioration of the patient's condition and an increased risk of OHCA. Cardioversion was used similarly frequently (*p* = 0.914) by both types of teams, but significant differences in the use of electrostimulation should be analyzed in further studies. The risk of OHCA in the course of ACS is high. The latest ERC guidelines emphasize management recommendations that include modifications to resuscitation procedures for patients after cardiac surgery, as well as in the angiography laboratory (31). In the prehospital setting, a solution that can significantly increase patient survival is the ECPR (extracorporeal cardiopulmonary resuscitation) procedure, which is still being developed in Poland.

### 4.4 Limitations of the study

Include failure to include the full number of ground-based emergency medical teams. The authors thus enabled the correlation of two groups (EMS vs. HEMS) with identical medical competencies with a doctor on board, yet an analysis of all ground team missions to ACS cases could provide a broader perspective on the issue at hand. Despite the use of digital databases, some data gaps made it necessary to limit the number of interventions analyzed. Attention should also be paid to combined interventions, where a HEMS team is called by a ground team due to, e.g., a significant distance from a higher referral-level hospital. This is when the EMS team implements the initial treatment, even before HEMS arrives, which can affect the overall statistics. While differences between EMS and HEMS are reported, the manuscript does not sufficiently address confounding factors, such as: patient severity at dispatch, geographic and logistical differences, time-to-treatment intervals, selection bias in HEMS activation.

## 5 Conclusions

The profile of ACS patients from the perspective of emergency medical teams indicates women with an average age of 66 years and diagnosed with I21 according to the ICD-10 classification. There are significant differences in the behavior of EMS and HEMS physicians. Both types of EMS teams were observed to be more likely to administer morphine than fentanyl, which contradicts recent medical knowledge suggesting a preference for fentanyl due to its more favorable action profile. Ground team patients are significantly more likely to receive oral P2Y12 receptor antagonists. HEMS teams, on the other hand, were more likely to administer muscle relaxants, dopaminergic drugs, and adrenergic drugs. External electrical stimulation was also more commonly used in HEMS for hemodynamically unstable patients. Future studies are worth considering to understand the correlation between the performance of these procedures by EMS physicians before hospitalization. Consideration should be given to standardizing the use of fentanyl instead of morphine in national prehospital protocols for patients with ACS.

## Data Availability

The datasets presented in this article are not readily available because of the non-public database of air ambulance services in Poland. Requests to access the datasets should be directed to piotr.leszczynski@uws.edu.pl.
